# Characterizing grazing and terrain use patterns of Hispano‐Breton mares in the Spanish Pyrenees using GPS devices and remote sensing data

**DOI:** 10.1111/avj.70014

**Published:** 2025-09-04

**Authors:** J Plaza, N Sánchez, JA Abecia, J Nieto, F Canto, ME Pérez‐García, C Palacios

**Affiliations:** ^1^ Faculty of Agricultural and Environmental Sciences University of Salamanca Salamanca Spain; ^2^ Institute of Research in Environmental Sciences of Aragón (IUCA) University of Zaragoza Zaragoza Spain

**Keywords:** GPS, grazing patterns, mares, remote sensing, topography

## Abstract

Geotechnologies, such as Global Navigation Satellite Systems (GNSS) and remote sensing, are essential for documenting topographic features and analyzing land use. Among them, the GPS (Global Position System)‐based sensors have proven highly effective in monitoring livestock, providing high‐resolution data on movement patterns. This study tracked two Hispano‐Breton mares in the Spanish Pyrenees during summer 2023 using GPS collars. A°C (LiDAR) dataset provided the digital elevation model (DEM), while Sentinel‐2 imagery assessed the grazing conditions. All data were integrated within a Geographic Information System (GIS). The study period ranged from 1 July to 28 August 2023. Until 7 August, the mares grazed in a valley area, while from that date on they traveled to high mountain pastures. The mares and their foals traveled a mean distance of 472.99 km, averaging 7.95 ± 2.58 km per day with a mean elevation gain of 561 m daily. Distance traveled increased with elevation gain, likely to mitigate steep slopes. Normalized Difference Vegetation Index (NDVI) analysis revealed that lower valley pastures maintained stable vegetation throughout the season, whereas high mountain pastures became significantly drier in August. These findings suggest that equine grazing patterns are shaped by forage availability, and possibly also by traditional herding practices. Although this study focuses on Hispano‐Breton mares in the Spanish Pyrenees, the results provide insights applicable to horses managed in extensive grazing systems worldwide, including wild and feral populations in arid and semi‐arid regions such as the Australian outback. Notably, the movement patterns observed in this study more closely resemble those of Australian domestic horses confined to large paddocks than those of feral horses, despite our mares being part of free‐range grazing systems. This study highlights the joint value of GPS tracking and remote sensing in understanding equine behavior in mountainous environments, offering insights for sustainable husbandry practices in high‐altitude regions.

**Abbreviations**
DEM: Digital Elevation Model
GIS: Geographic Information System
GNSS: Global Navigation Satellite Systems
GPS: Global Position System
LiDAR: Light Detection and Ranging
NDVI: Normalized Difference Vegetation Index
PLF: Precision Livestock Farming
PNOA‐LiDAR: Spanish National Plan for Aerial Orthophotography
UTM: Universal Transverse Mercator

Livestock farming has undergone significant transformations and is now deeply embedded in the ongoing agricultural and livestock technological revolution. Within this evolving landscape, precision livestock farming (PLF) has emerged as a novel paradigm. PLF utilizes devices such as sensors and actuators, along with corresponding methodologies, to collect real‐time data aimed at optimizing farm management systems.^1^ It can also be defined as a farming approach that employs equipment, data, or software to leverage individual‐based information for more precise management decisions, input applications, and treatments.^2^

Technological advancements in the livestock sector have made data collection and analysis essential tools for strategic decision‐making. The integration of PLF has enhanced knowledge and improved the ability to predict and respond to various factors affecting production performance.^3^ This enables producers to optimize resource use, enhance animal health and welfare, and reduce the environmental impact of livestock production.^4^ Its application has expanded to various livestock species, including horses, facilitating studies on behavior, such as location tracking with GPS and movement detection using accelerometers,^5^ as well as early disease detection, ultimately contributing to improved animal welfare.^6^

For over two millennia, seasonal movement of cattle, sheep, and horses have been widely practiced across the Old World, with particular prominence in the Iberian Peninsula. This region is characterized by highly variable climatic and geographical conditions that enable high biomass production in different locations and at different times of the year.^7^ As a result, migratory systems are essential for the efficient use of an ecosystem's primary productivity, allowing access to resource‐rich areas at different times^8^ and optimizing the use of grazing resources.^9^ These movements not only support ecosystem regeneration but also help maintain a balanced carrying capacity of the land, preventing overexploitation of specific areas and promoting forage renewal.^10^

Another important aspect of livestock movement is that, despite reaching its peak in recent decades, it remains a socioeconomic pillar for some farmers.^11^ Although seasonal movement of livestock has declined drastically, primarily due to intensification and a lack of generational succession, it still holds significant cultural value, particularly in rural areas. In these regions, there is continued interest in preserving these practices, as they contribute to rural development and foster a sustainable relationship with the environment.^12^ Moreover, understanding the behavioral patterns of grazing animals and the key factors influencing their decision‐making when selecting grazing areas, such as topography and forage availability, is essential.^13^

Currently, there is limited knowledge about the foraging behavior of free‐ranging animals in highly biodiverse grasslands. In this context, PLF emerges as a key tool for analyzing and understanding these patterns, allowing for the inference of feeding habits and the optimization of management strategies.^14^ Geotechnologies have the potential to significantly enhance PLF by providing information on topographic attributes and the biotic composition of the terrain. They also allow for tracking animal geolocations and walking times through miniaturized wearable devices. Among these technologies, Global Navigation Satellite Systems (GNSS), particularly GPS‐based devices mounted on collars, have proven highly valuable for tracking and monitoring cattle,^15^, ^16^ studying the behavior and circadian rhythms of grazing sheep,^13^, ^17^ and measuring distances traveled by horses.^18^, ^19^ To assess terrain attributes such as contour, slope, and aspect, the geolocations recorded by these devices can be seamlessly integrated into GIS and overlaid onto reference maps, including DEM or orthophotographs.^20^, ^21^, ^22^ Additionally, to characterize vegetation cover, remote sensing imagery from satellite missions such as Landsat or Sentinel‐2 can serve as a foundational layer in GIS analyses for generating vegetation indices,^23^ including the widely used NDVI. Despite recent advancements, the integration of cutting‐edge remote sensing technologies like LiDAR remains relatively unexplored, likely due to the historically limited availability of free datasets. Nevertheless, previous studies^24^, ^25^ have demonstrated that LiDAR data offer valuable insights for analyzing both terrain features and vegetation structure.

In Australia, the movement and grazing patterns of feral horses in the outback have been studied using similar GPS‐based methodologies, providing valuable insights into the behavior of horses in extensive grazing systems. Our study, although focused on Hispano‐Breton mares, shares clear parallels with this Australian research, particularly in how terrain and forage availability influence grazing decisions.^18^, ^26^

Therefore, this study focused on monitoring two 6‐year‐old adult Hispano‐Breton mares in the southern Pyrenees using two DMS‐CattleSat 1.4 GPS devices. The study period spanned from 1 July to 28 August 2023. The research had two primary objectives. First, it aimed to determine the distances traveled by the mares, the slopes they traversed, average journey metrics, and gradients. Second, it sought to characterize the grazing areas selected by the mares using NDVI data from a Sentinel‐2 time series throughout the summer of 2023.

## Material and methods

### Study area and calendar

**Figure 1:**
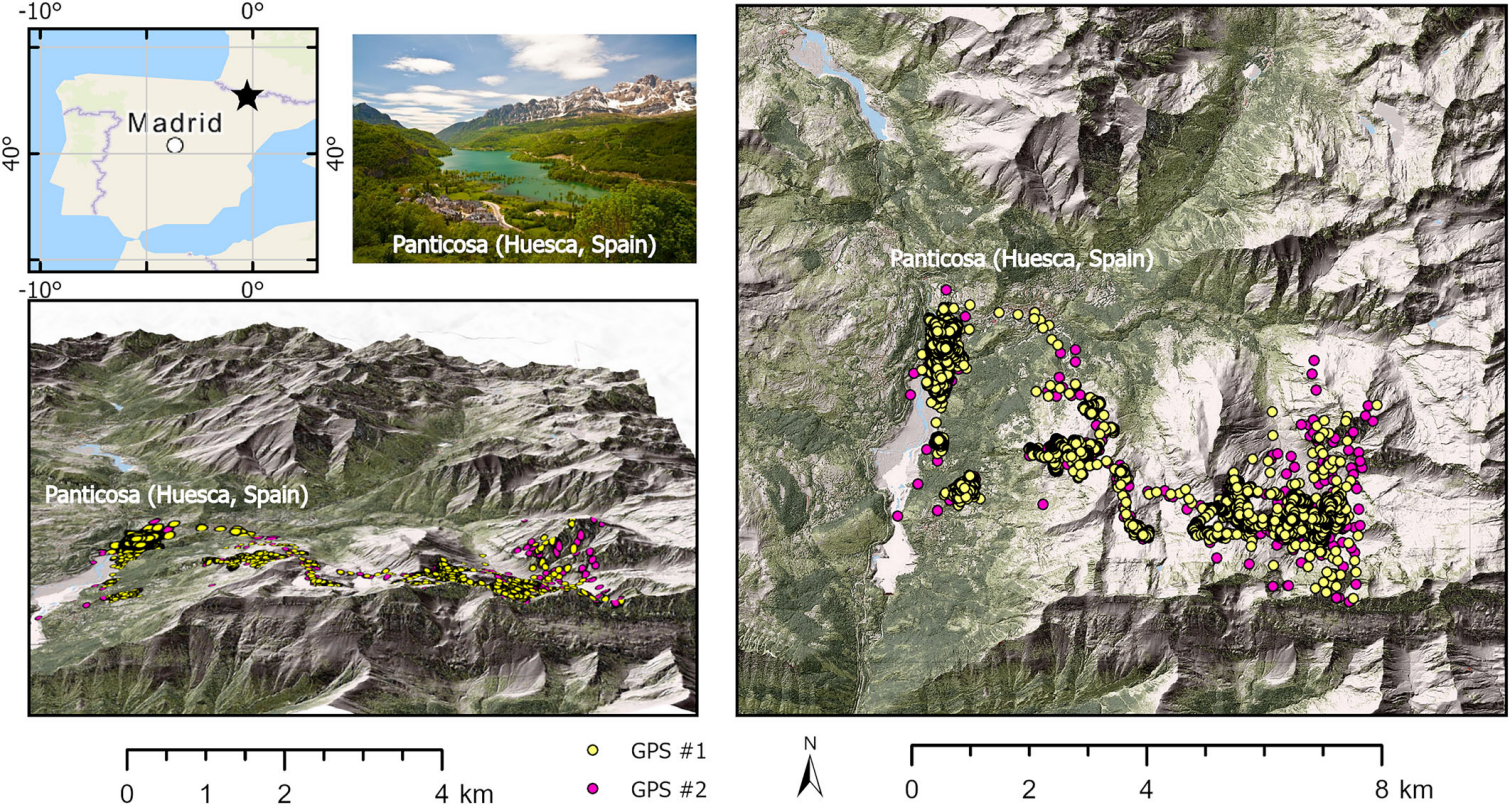
Study area and GPS positions of the mare herd.

This study was conducted in the Spanish Pyrenees (Figure 1), covering different mountainous landscapes, specifically distinguishing between valley terrain (Panticosa, Huesca, Spain; 1184 m a.s.l.) and high‐altitude pastures (2380 m a.s.l.). The mares had free access to this entire elevational range, as they moved unrestricted across the landscape. This mountain range serves as a climatic transition zone, influenced by both the oceanic conditions of the Navarran Pyrenees and the Mediterranean climate of the eastern Catalonian valleys. In the central Spanish Pyrenees, temperature and precipitation vary depending on altitude and proximity to the Atlantic Ocean and Mediterranean Sea. On average, temperature decreases with elevation at a rate of approximately 0.6°C per 100 meters.^27^ In the Panticosa valleys, the mean annual temperature is 6.4°C, with an average annual precipitation of 1280 mm, according to data from the Spanish Meteorological Agency. The warmest month is July, with an average temperature of 14.8°C, while February is the coldest, averaging 0.1°C. Rainfall is lowest in July (74 mm) and highest in November (154 mm).

At higher altitudes, summer pastures experience different climatic conditions, with a mean annual temperature of 8.1°C. January is the coldest month, averaging 1.5°C, while July is the warmest at 17.1°C. Total annual precipitation reaches 1025 mm, with the lowest recorded in May (26 mm) and the highest in November (216 mm). Snow cover persists from November to April, limiting vegetation growth and reducing the abundance of legume species.^28^

The landscape is predominantly composed of pastures and grasslands, with only 3% covered by scattered tree patches. The subalpine mountain grasslands of the Central Spanish Pyrenees are semi‐natural ecosystems shaped and maintained by human activity, with their long‐term sustainability closely tied to grazing practices.^28^

### Animal data

The study focused on two adult Hispano‐Breton nursing mares, aged 6 and 8 years, respectively, with foals typically born around May. These mares formed part of a small social group of four individuals that remained together throughout the study period. The Hispano‐Breton heavy horse breed originated in the 1930s through the crossbreeding of Breton stallions, primarily introduced in the Cornisa Cantábrica, the Pyrenees, and the mountainous regions of Castilla y León, with local mares.^29^ This breeding program aimed to enhance the robustness of Spanish horses.^30^ Today, the breed benefits from special legal protection due to its endangered status,^31^ primarily attributed to agricultural mechanization.

Hispano‐Breton horses are typically recognized by their brown or chestnut coats, rectilinear facial profiles, and highly muscular necks. Males of this breed weigh an average of 715 kg, while females average 702 kg. These horses are managed under extensive grazing systems, with mares and their foals grazing together in both valley and high mountain pastures for most of the year.^29^ Breeding occurs through natural mating, with efforts to concentrate foaling between March and June. Although the productive role of Hispano‐Breton horses has evolved over time, their primary purpose today remains foal meat production.

Traditionally, the herd follows a traditional grazing cycle, spending the summer months (July to September) in high mountain pastures before returning to valley lands for the rest of the year. This management strategy is not solely based on the availability of forage and free‐grazing principles but also considers various economic and social factors. These may include profitability, tourism activity, the presence of insects near water reservoirs, and other relevant conditions influencing movement decisions.

### GPS devices

**Figure 2:**
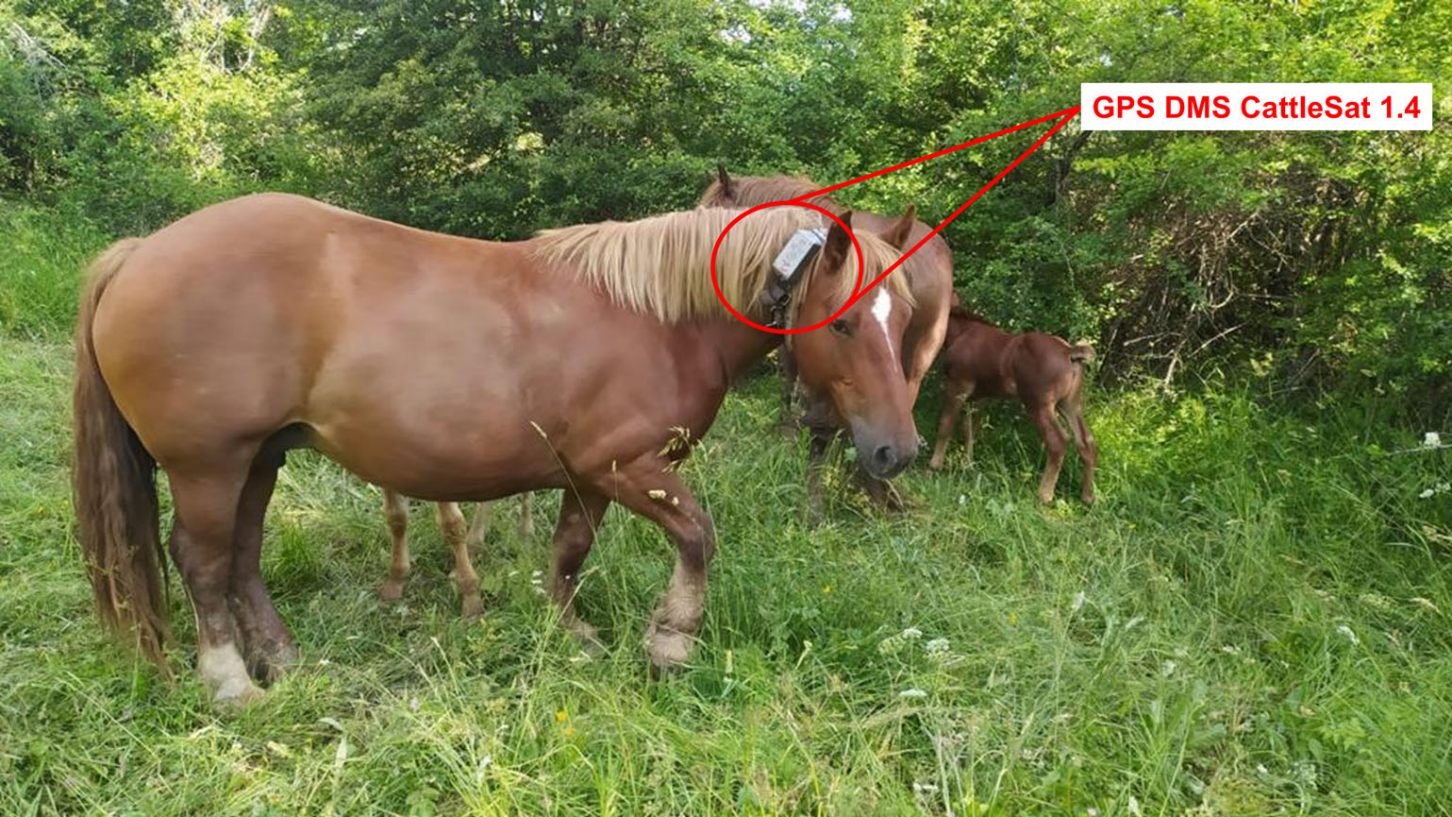
GPS collar mounted in a Hispano‐Breton mare. Detail of the equipment.

On 1 July, GPS collars were fitted onto two randomly selected, healthy mares (Figure 2). The tracking system employed a DMS‐CattleSat 1.4 GPS sensor paired with a UBX‐R3 receiver chip (Domodis, Cordovilla, Spain), recording the geographic coordinates (latitude and longitude) of each mare's position approximately every 5 min. To conserve battery power, data collection was paused when the animals remained stationary for more than 5 min. The system maintained a location accuracy error of less than two meters.

All components, including the GPS sensor, long‐life battery, and satellite signal‐receiving antenna, were encased in a durable, hermetically sealed plastic housing (115 × 64 × 40 mm) designed to protect against shock and moisture (Figure 2). The complete collar weighed 290 g, representing a non‐invasive method commonly used for monitoring livestock movements.

GPS data were retrieved from the Domodis tracking system (https://www.loc.gpsganado.es/), which generated a text file for each device. After reviewing and refining the dataset, coordinates were converted into Universal Transverse Mercator (UTM) format (X, Y). Since the GPS receiver did not record altitude (Z coordinate), elevation data were derived by overlaying geolocations onto an external 3D data source, as described in the next section. Using the resulting (X, Y, Z) dataset, calculations were performed to extract daily and cumulative values for distance traveled, elevation change (Z increment), and slope.

### Imagery

**Figure 3:**
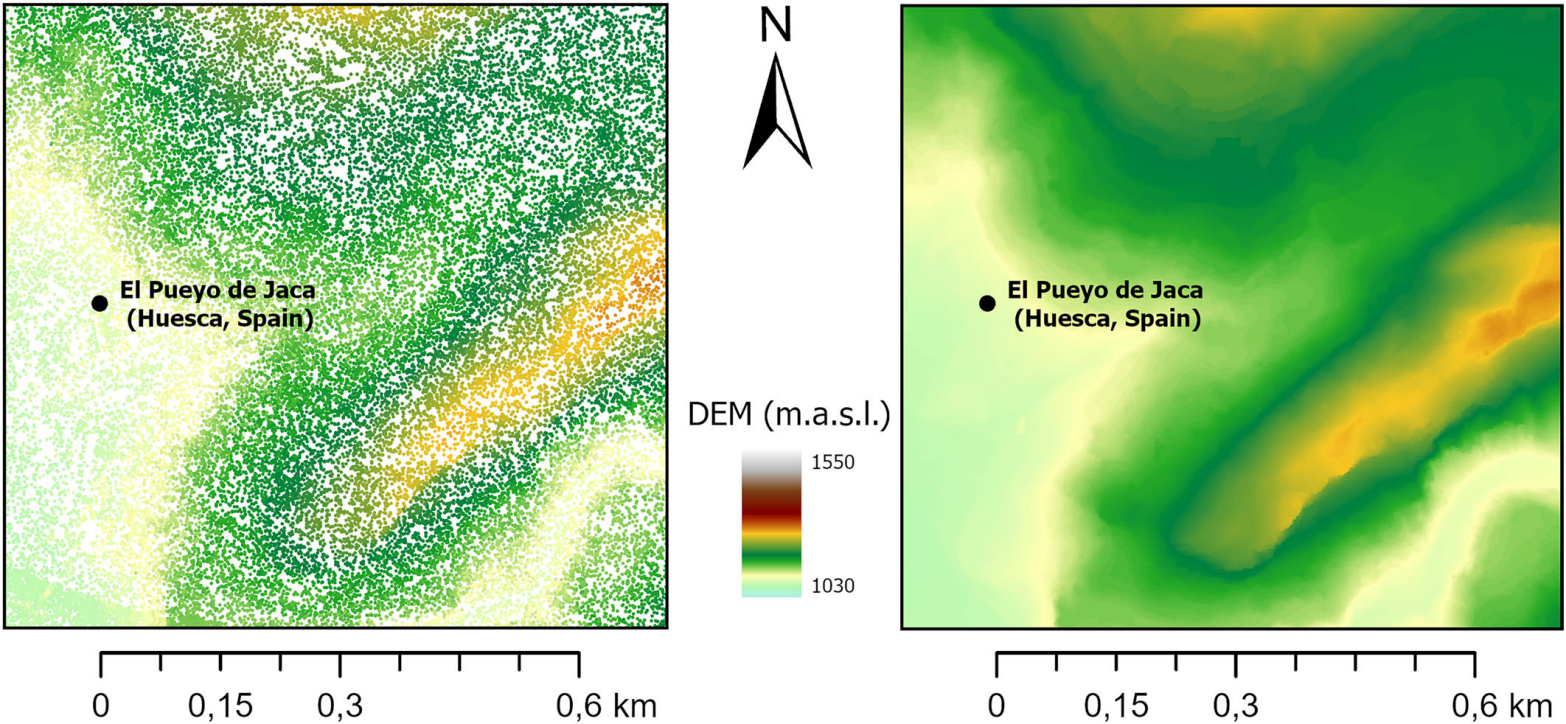
Detail of LiDAR point cloud (left) and LiDAR‐derived raster DEM (right).

To determine the Z coordinate of each mare's location, an airborne LiDAR dataset was used to generate a DEM of the study area. LiDAR employs active laser sensors to generate a dense point cloud of the Earth's surface. Specifically, it emits laser pulses toward the ground and records both the amount of energy reflected back and the time taken for its return, allowing for precise topographic and vegetation structure mapping.^13^ The dataset comprised 18 tiles (2 × 2 km each) from the 2021 Spanish National Plan for Aerial Orthophotography (PNOA‐LiDAR), with a resolution of 0.5 to 22 points/m^2^.^32^, ^33^ These datasets have a nominal accuracy of 25 cm for the Z coordinate and 50 cm for XY coordinates.^34^, ^35^ The LiDAR tiles were mosaicked, filtered, and converted into a raster image with a 1 × 1 m spatial resolution. Subsequently, the XY locations of the mares were overlaid onto the raster DEM to extract the corresponding Z values (Figure 3). All geoprocessing was performed using ArcGIS Pro 3.0.

To assess the vigor of the grazing areas preferred by the mares, a time series of Sentinel‐2 remote sensing images was used to analyze vegetation conditions. The Sentinel‐2 mission, part of the European Copernicus satellite program,^36^ monitors land surface conditions such as vegetation cover and land use. The constellation consists of twin satellites, Sentinel‐2A and Sentinel‐2B, both equipped with multispectral imagers capturing 13 spectral bands in the visible, near‐infrared, and shortwave infrared ranges. A total of six cloud‐free Sentinel‐2A and 2B images were downloaded and processed. The NDVI was computed from the red and near‐infrared bands (10 m spatial resolution), resulting in an NDVI time series for the following dates: 24 June 2023, 14 July 2023, 27 July 2023, 8 August 2023, 11 August 2023, and 31 August 2023.

NDVI is closely associated with vegetation vigor and density^37^ and was therefore used in this study as a proxy for assessing pasture freshness and productivity. This allowed for an evaluation of the mares' spatial preferences for grazing and movement. To analyze pasture health across the study area, all mare locations were overlaid onto each NDVI dataset.

Given the presence of two distinct topographic scenarios, the lower valley with gentle slopes and the rugged, stepped terrain of the high mountains, further analysis was conducted. Using 7 August as a reference date marking the transition between these two grazing zones, the GPS locations were divided into two groups: those recorded before and those recorded after this date. The average NDVI value was then calculated for each dataset.

## Results

### Distance, elevation, slope

The mares travelled a total distance of 472.99 km, with an average daily movement of 77.95 ± 2.58 km (Table 1), ranging from 2.32 to 15.27 km per day. All 40 mares in the group were lactating, which reinforced their gregarious behavior. Therefore, it could be inferred that the GPS data from the two individuals are representative of the group's movement patterns. Over the course of the study, the cumulative elevation gain and loss totaled 33.38 km (in absolute values), with a daily elevation change of 561 m. Given that the study area is characterized by steep mountainous terrain, ranging from 1,184 m a.s.l. to 2,380 m a.s.l., these movement patterns are particularly noteworthy.

**Table 1:** Distance, elevation (Z increment) and slope (absolute values) traveled by two mares monitored with GPS sensors in the Spanish Pyrenees during the summer

GPS collar	Total distance (m)	Average distance/day (m)	Total Z increment (m)	Average Z increment/day (m)	Average slope/day (%)
#1	463,920	7732 ± 2.283	34,845	581	7.63
#2	482,050	8170 ± 2.885	31,925	541	8.15

**Figure 4:**
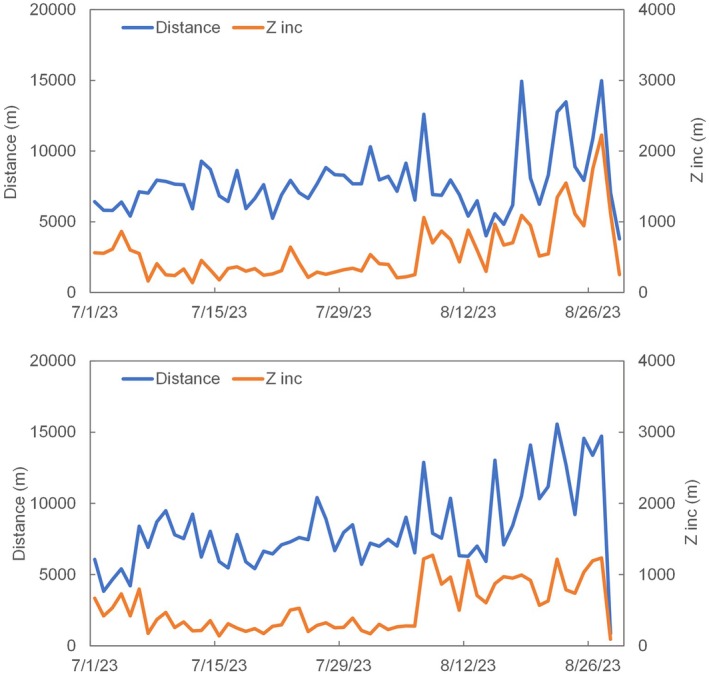
Distance and Z increment (daily) traveled by the mares. Collar #1 (top), collar #2 (bottom).

The daily distance travelled and elevation gain (Figure 4) show an increasing trend as the mares moved from lower valleys to higher peaks. Both variables follow a similar pattern, with a strong Pearson correlation (R = 0.61) between distance covered and elevation gained. As shown in Figure 3, the highest daily elevation gains precisely align with the longest distances travelled each da. This effect leads to a moderate average slope of approximately 8%, thereby smoothing the ascent.

### Spatial and temporal pattern of grasslands

**Figure 5:**
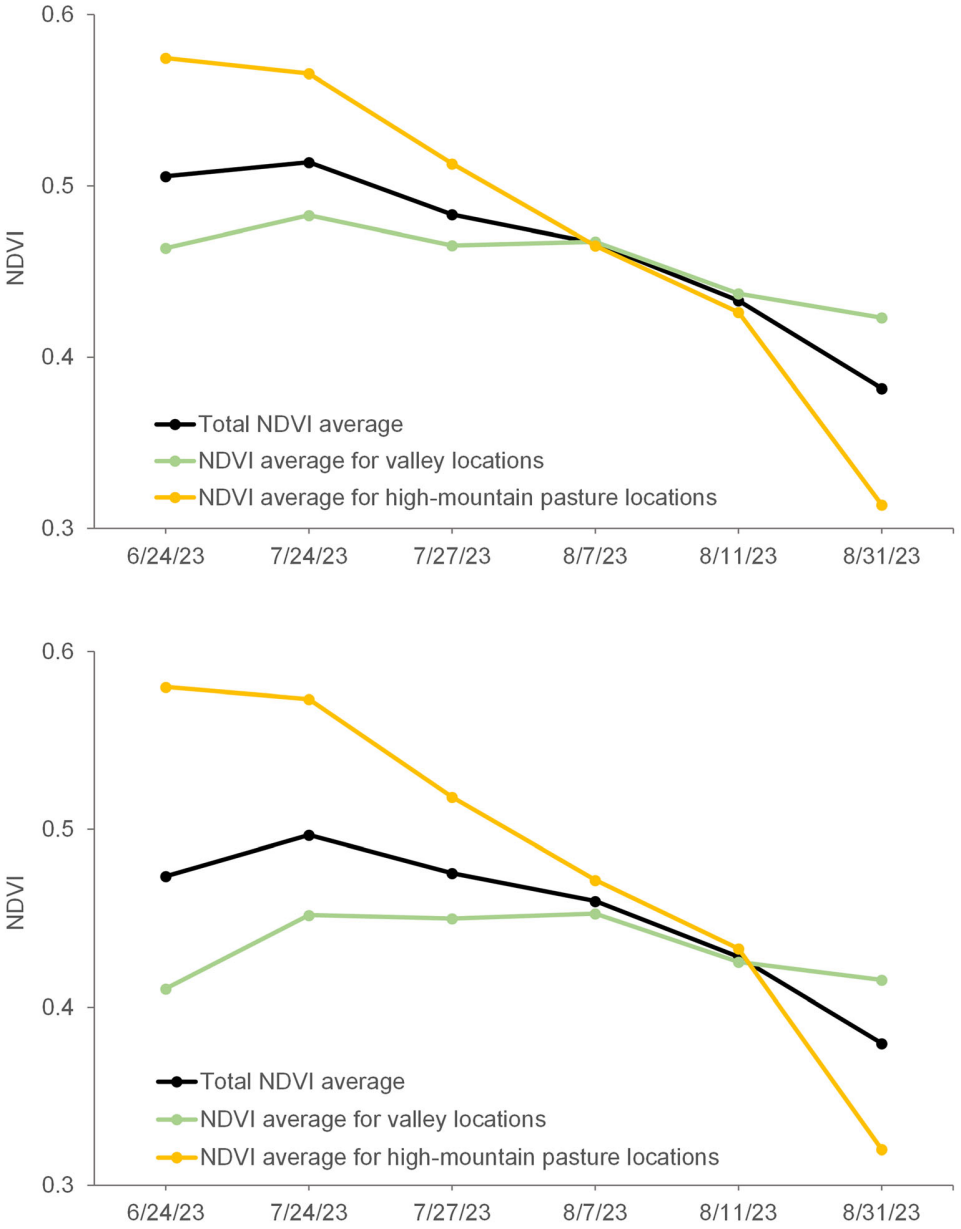
Averaged Normalized Difference Vegetation Index (NDVI) of the GPS locations from the Sentinel‐2 series. Collar #1 (top), collar #2 (bottom). The green line corresponds to GPS locations before 7 August (valley pastures), whereas the yellow line corresponds to GPS locations after 7 August (high‐mountain pastures).

The average NDVI values recorded at each Sentinel‐2 date for both GPS collars exhibited a remarkably similar trend (Figure 5). It should be noted that NDVI was monitored throughout the entire study period in both study areas to assess the evolution of pasture conditions before, during, and after the presence of the animals. A noticeable decline in NDVI at the beginning of August indicated that grasslands were drying out, reflecting the prevailing climatic conditions in the study area.

The NDVI pattern varied depending on livestock occupancy and the period considered. When the mares were located near the valley reservoir, pasture conditions (in terms of NDVI) remained relatively stable between July and August (Figure 5, green line). This suggests that lower valley areas maintain consistent vegetation vigor throughout the season. By contrast, the higher, more rugged terrain mountain grassland conditions were notably better in July than in August (Figure 5, yellow line).

Contrary to the aforementioned temperature conditions, in these upper areas, temperatures are likely slightly higher than in the valleys (17.1°C *vs*. 14.8°C) due to increased solar radiation exposure and the absence of the reservoir's microclimatic influence. As a result, high‐altitude pastures experience drier conditions in August, with NDVI values around 0.3 to 0.44, whereas, they are more productive in July, maintaining higher NDVI values around 0.6. This pattern was consistently observed in the data from both GPS collars (Figure 5).

## Discussion

In this study, geotechnologies enabled a precise characterization of the routes taken by Hispano‐Breton mares in the Pyrenees, considering both topographical features and vegetation cover condition along their path. These data provide valuable insights into the movement dynamics of equids in mountainous landscapes and their interaction with available forage resources. These findings support the development of more effective and sustainable management strategies for high‐altitude equine husbandry, reinforcing the viability of extensive grazing systems in such challenging environments.

Although PLF studies often involve a limited number of animals, the focus is on analyzing the extensive datasets generated, which can be representative of the overall group's behavior.^38^ In this context, despite tracking only two mares, each GPS device recorded over 12,000 geolocations throughout the study period, yielding a robust dataset for assessing grazing behavior in both valley and high‐mountain pastures. Additionally, considering the homogeneity among the 40 mares, exhibiting similar weight, body condition, age, and physiological state (all with foals).

The results of this study are in close agreement with those of Hampson et al.,^18^ who reported that mares with foals traveled 7.2–7.6 km/day when grazing in large paddocks. The distance traveled by Australian wild horses was longer than that of our Hispano‐Breton mares, which may be attributed to the mountainous terrain, availability of water or specific breed characteristics.^26^ Therefore, the movement patterns observed in our mares more closely resemble those reported for domestic horses grazing in small paddocks^18^ than those documented for feral horses in the Australian outback.^26^ Such parallels make our findings directly relevant to Australian veterinarians and livestock managers seeking to improve equine welfare and management in extensive systems.

Additionally, the pattern observed in this study, where animals travel longer distances to reduce ascent in areas with steep slopes, is consistent with the findings of García‐González et al. (1990),^39^ who noted that free‐grazing sheep and cows in the Pyrenees also avoid steep slopes and rocky ridges, preferring routes with gentler gradients. These results reinforce the idea that movement patterns of herbivores in mountainous environments respond to strategies for optimizing energetic expenditure as a function of relief and forage availability.

Similarly, this phenomenon aligns with the assumption tested by Ganskopp et al. (2000)^40^ that livestock establish pathways of least resistance between frequently visited areas of their pastures. These authors discovered that livestock opted for cross‐slope routes, effectively reducing the slope traversed from 13.5% to 8%, a finding closely mirroring the results of this study, developing lower effort travel routes in hilly terrain. A plausible explanation is that as elevation gain increases, the horizontal distance required to ascend also rises, resulting in a reduced slope ratio.

Interestingly, Spanish folklore suggests that donkeys (and by extension, mares) possess an innate ability to instinctively select the optimal path in steep terrain.^41^ In mountainous areas, livestock seek a balance between obtaining feed and conserving energy by choosing pastures located in flatter areas where there is less effort to move.^42^ This strategy allows animals to maximize their energy efficiency while still accessing adequate feed resources. In free‐grazing systems, animals show a clear preference for moving on slopes of less than 20%, as steeper slopes require more energy expenditure.^43^

Traditionally, livestock is driven towards higher mountain areas during the hot season, which typically offer cooler and sun‐sheltered environments.^29^, ^44^ However, as demonstrated here, these areas may exhibit lower pasture quality or vigor. In this particular case, the presence of a water reservoir (a man‐made lake) in the valley region significantly altered these conditions, providing the animals with a cooler and a more stable environment than the high‐mountain pastures.^45^ This discrepancy may be attributed to the valley's milder and more stable temperature and precipitation conditions compared with the high‐mountain pastures, which may experience harsher and more variable microclimates.

Such observations challenge conventional equine husbandry practices in mountainous regions, which are increasingly driven by logistical factors rather than pasture condition and availability. For instance, livestock farmers tend to avoid tourist‐populated valleys to protect their animals from the disturbance caused by constant human presence. Consequently, tourism in this area negatively impacts extensive livestock farming, disrupting the productive activity of livestock farmers.^46^ Additionally, farmers often move their horses to higher altitudes, particularly during periods of high insect abundance. This practice not only shields the animals from fly‐borne discomfort and disease but also contributes to improving their overall welfare.^47^

Therefore, the migration of the herd to the higher regions might be uniquely triggered by logistic factors considered by the herder, as previously mentioned. Ironically, during these periods of poorer pasture conditions in August, mares and foals undertake greater efforts, including longer distances, and steep ascents.

## Conclusions

In conclusion, the integration of GPS devices and remote sensing data proved to be effective in documenting the movements of the study herd of Hispano‐Breton mares and their foals as they moved from the valleys of Panticosa to the high mountain pastures in the Spanish Pyrenees. The Hispano‐Breton mares covered daily distances of 7.95 km and ascended 561 m on average. Additionally, the NDVI analysis revealed distinct pasture conditions between the higher mountain areas, which were drier in August but more productive in July, and the more stable conditions in the Panticosa valley, likely influenced by the presence of the water reservoir (a man‐made lake). This finding suggests that, in the Spanish Pyrenees, conventional equine husbandry practices may be more shaped by logistical decisions made by the farmer than by pasture quality and availability under specific circumstances.

## Conflicts of interest and sources of funding

The authors declare no conflicts of interest or sources of funding for the work presented here.

## Data Availability

The data that support the findings of this study are available from the corresponding author upon reasonable request.
